# Eosinophilic Sialodochitis Mimicking Sjögren’s Syndrome

**DOI:** 10.7759/cureus.87820

**Published:** 2025-07-13

**Authors:** Athira Sasidharan, Stuart Carter, Hannah Walsh, Christopher Platais

**Affiliations:** 1 Rheumatology, Sheffield Teaching Hospitals, Sheffield, GBR; 2 Oral and Maxillofacial Pathology, Sheffield Teaching Hospitals, Sheffield, GBR; 3 Oral Medicine, Sheffield Teaching Hospitals, Sheffield, GBR

**Keywords:** eosinophilic sialodochitis, igg4 disease, kussmaul disease, salivary gland swelling, sjögren’s disease

## Abstract

Eosinophilic sialodochitis (ES) is a rare allergic condition affecting the major salivary glands. It may present to rheumatologists by mimicking Sjögren’s syndrome. ES typically presents with recurrent swelling of the major salivary glands and oral dryness due to low-quality, low-volume saliva production. Although the condition is not completely understood, the most accepted aetiology of ES is an allergic process, which is characterised by eosinophilic predominant infiltration of salivary gland tissue, eosinophilic-rich mucous plugs and peripheral eosinophilia. We present a rare case of ES mimicking Sjögren’s syndrome.

## Introduction

Eosinophilic sialodochitis (ES), also known as ‘sialodochitis ﬁbrinosa’, ‘Kussmaul disease’, ‘allergic parotitis’, ‘chronic sialodochitis with eosinophilia’, ‘idiopathic bilateral salivary mega canal’, ‘sialodochitis with eosinophilic inﬂammation’ and ‘idiopathic eosinophilic parotitis’, was first reported in 1879 by Kussmaul to describe idiopathic recurrent parotid gland swelling with mucus plugs laden with leukocytes and Charcot-Leyden crystals [1]. The aetiology of ES is still not understood, although the prevailing hypothesis suggests an allergic process. Baer et al. revealed a marked female predominance (2.3:1), median age being 47 years, a prevalence of atopy at 63% and peripheral eosinophilia of 71% [1]. A diagnosis of ES should be considered when the patient presents with salivary gland swelling [2]. Here, we present a case of eosinophilic sialodochitis, showcasing the clinical presentation, workup strategy and clinical response to treatment. In this case, the patient’s symptoms were chronic and accompanied by dry mouth and itchy eyes, leading to a rheumatology referral, where we considered alternative differential diagnoses including Sjögren’s syndrome, sarcoidosis, sialosis and immunoglobulin G4 (IgG4)-related disease.

ES is a relatively rare lesion that should be considered in the differential diagnosis of recurrent major salivary gland swelling [1].

## Case presentation

A woman in her 40s was referred by colleagues in maxillofacial surgery to the rheumatology clinic due to a 10-year history of bilateral recurrent parotid swelling and pain, consistent with recurrent episodes of sialadenitis. Bilateral submandibular sialadenectomy in 2016 was a last resort treatment and was histologically reported as most likely IgG4 disease at the time. Investigations, including multiple sialendoscopies, had demonstrated chronic sialadenitis, with suspicion of IgG4-related disease, due to two prior episodes of autoimmune pancreatitis. However, histopathological analysis from salivary gland tissue removal was not typical for IgG4-related disease but demonstrated fibrotic change in both salivary ducts and gland parenchyma consistent with chronic sialadenitis, lymphocyte and eosinophilic infiltration. Multiple salivary gland steroid injections were given to treat acute episodes, with only short-term benefit.

Typical symptoms included recurrent episodes of acute pain and swelling, progressing to chronic persistent pain beneath the jaw, which lasted for several hours most days, with some relief by massaging the area of swelling. Oral examination showed moist mucosa, and she did not report dryness as a symptom. Her main complaint was expressing strings of saliva from the salivary gland ducts, which is a classic symptom of ES. She had itchy eyes that improved with anti-allergy eye drops. 

**Figure 1:**
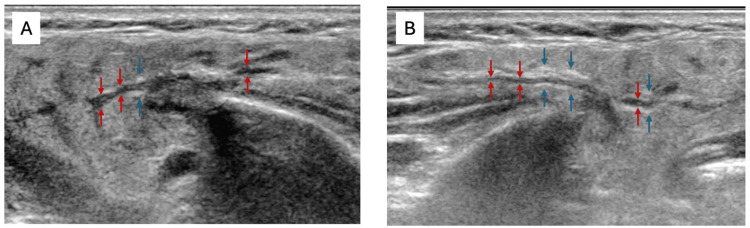
Ultrasonographic appearances of parotid salivary glands The right (A) and left (B) parotid glands show similar ultrasonographic appearances, with ductal dilatation extending throughout the gland (red arrows). The main dilated ducts are surrounded by thick hyperechoic tramlines, suggestive of fibrosis (blue arrows). The background gland parenchyma is almost normal.

Her blood tests showed negative antinuclear antibody, extractable nuclear antigen and IgG. IgG4 subclasses were normal throughout, whereas immunoglobulin E (IgE) was elevated, with an intermittent peripheral eosinophilia up to 1.2 × 10^9^/L observed on review of historical blood tests. Positron emission tomography-computed tomography (PET-CT) demonstrated salivary gland fluorodeoxyglucose (FDG) uptake, but no other concerns. Blood tests have been tabulated in Table 1. Ultrasound of the swellings demonstrated parotid glands that exhibited duct dilatation bilaterally, extending into dilated hilum, with thick hyperechoic tramlines surrounding the main intraglandular ducts (Figure 1).

**Table 1:** Blood investigations IgG: immunoglobulin G

Immunology screening tests	Results	Reference ranges
Extractable nuclear antigen	Negative	-
Antinuclear antibody (Hep2)	Negative	-
Centromere	<9 IU/mL	0-13 IU/mL
Rheumatoid factor	0.62 IU/mL	0.04-0.5 IU/mL
Eosinophil count	1.2 × 10^9^/L	0-0.45 × 10^9^/L
IgG subclasses		
IgG1	9.20 g/L	3.2-10.2 g/L
IgG2	3.00 g/L	1.2-6.6 g/L
IgG3	1.08 g/L	0.2-1.9 g/L
IgG4	0.99 g/L	0-1.3 g/L

**Figure 2:**
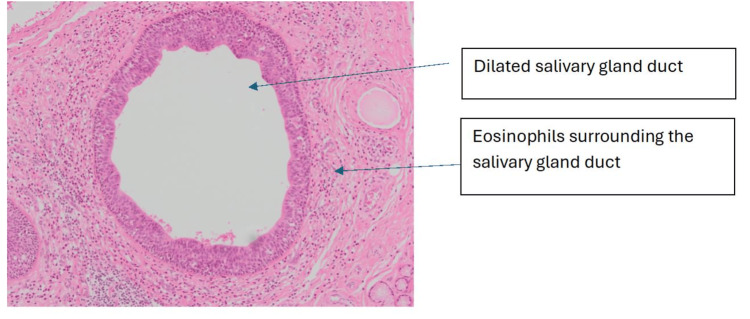
Haematoxylin and eosin-stained section at ×10 magnification: high-power image showing a dilated duct with collections of eosinophils present in a periductal distribution

**Figure 3:**
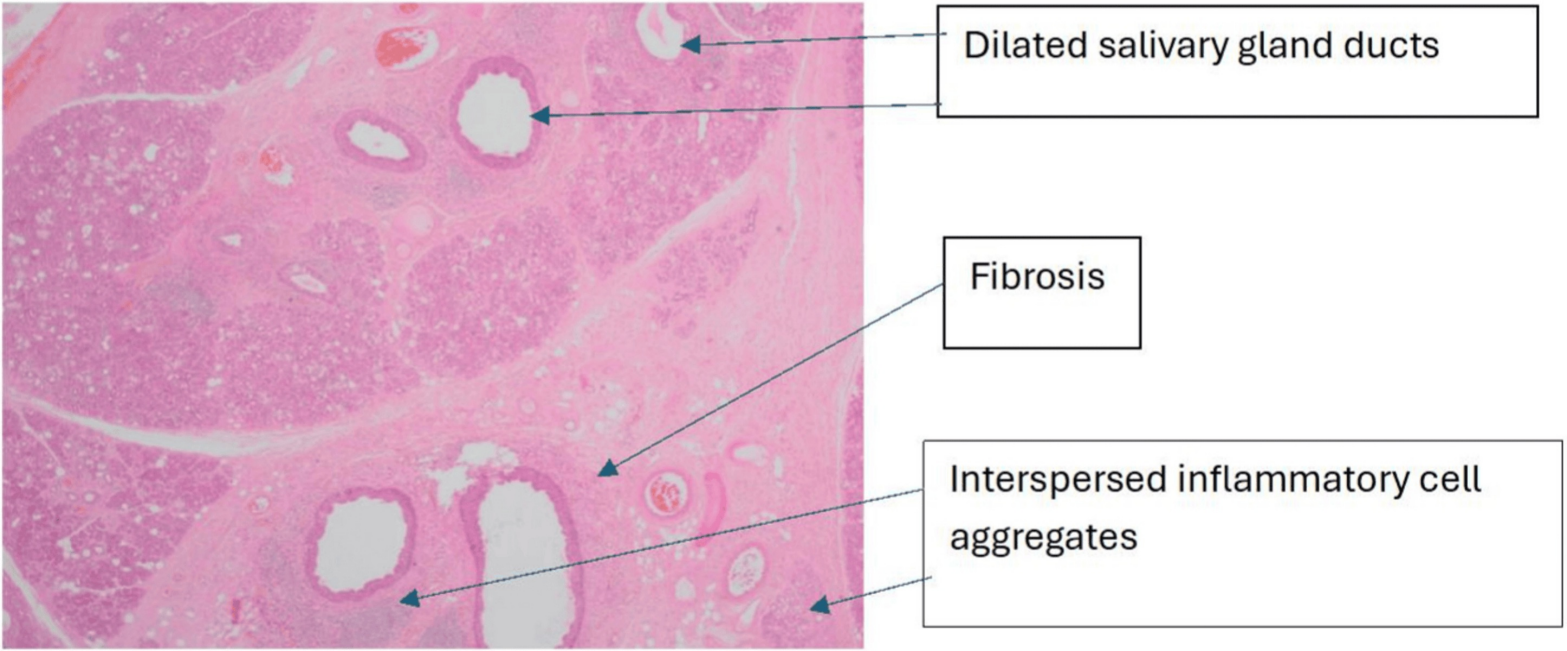
Haematoxylin and eosin-stained section at ×2 magnification: low-power image showing multiple dilated ducts with evidence of mild fibrosis, acinar atrophy and interspersed inflammatory cell aggregates

**Figure 4:**
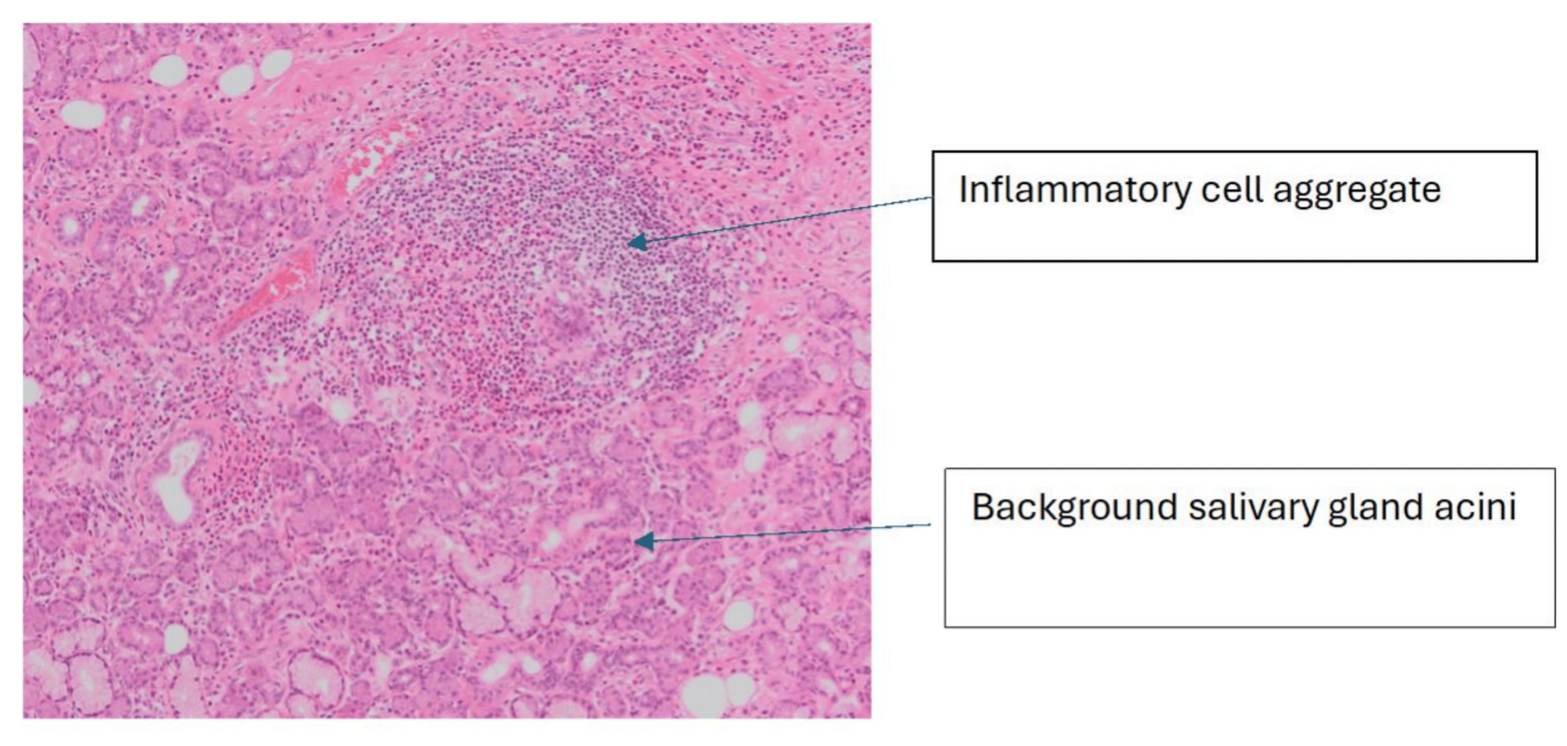
Haematoxylin and eosin-stained section at ×10 magnification: high-power image showing background acini with an inflammatory cell aggregate composed of predominantly lymphocytes and eosinophils

**Figure 5:**
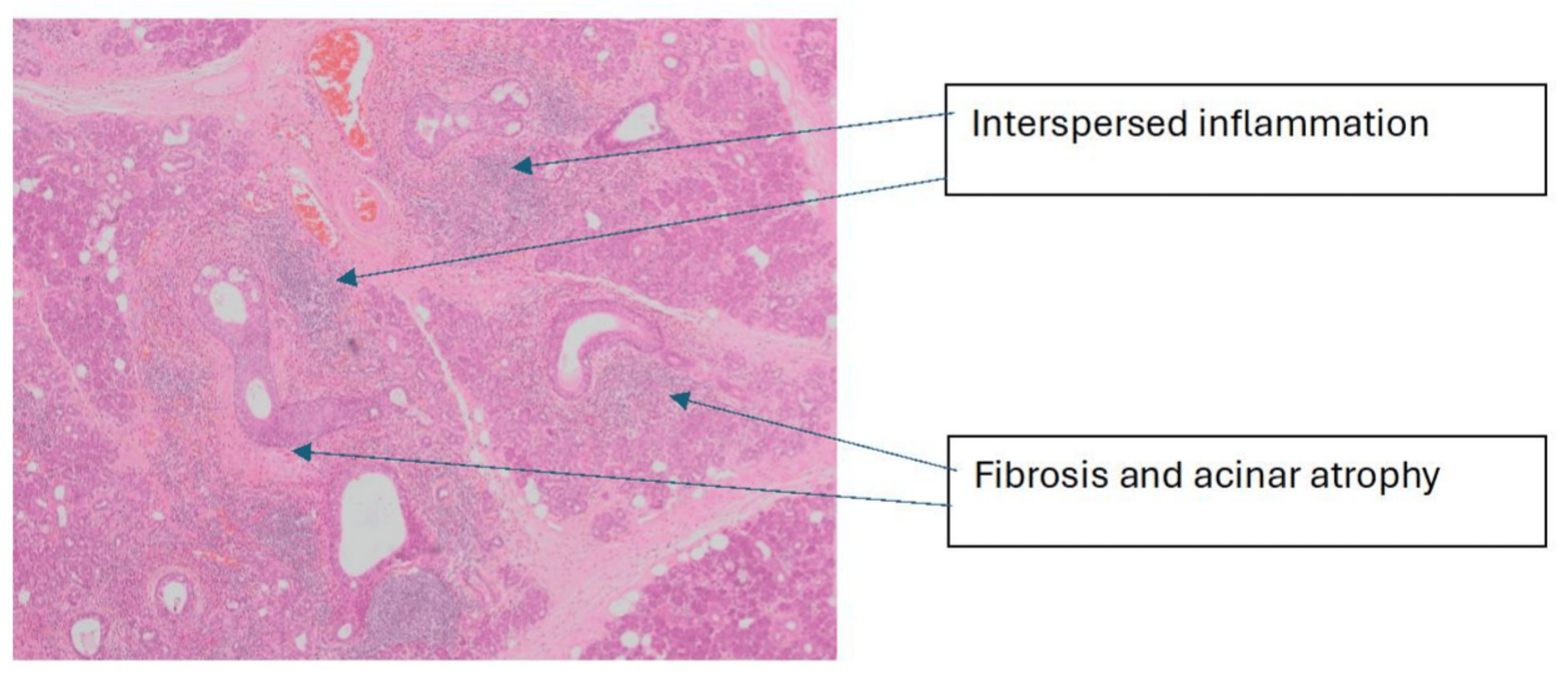
Haematoxylin and eosin-stained section at ×4 magnification: lower-power image showing mild fibrosis, acinar atrophy and aggregates of inflammatory cells interspersed between the acini

Given that there was a lack of diagnostic clarity, we re-examined the previously obtained salivary gland tissue. The histological analysis showed a mixture of mucous and serous acini with numerous dilated ducts throughout the glands. Mild fibrosis and acinar atrophy were observed, with focal aggregates of inflammatory cells interspersed between acini. Inflammatory infiltrate predominantly composed of lymphocytes was noted; however, occasional plasma cells were also observed, with an IgG to IgG4 ratio that did not satisfy the threshold to confirm IgG4-related disease. The eosinophilic infiltration in a periductal distribution was suggestive of eosinophilic sialodochitis. Histopathological images are depicted in Figures 2-5. Moreover, they were fitting into the criteria proposed by Baer et al., which helped us clinch the diagnosis. The patient was started on a combination of fexofenadine 180 mg daily and montelukast 10 mg daily, reporting improvement in symptoms of swelling and pain. She was subsequently followed up in the rheumatology clinic, where her symptoms had markedly improved with oral medications.

## Discussion

ES is a rare disease that was first described in 1879 by Kussmaul [1]. Previous studies have shown that the disease can occur in patients between the ages of 2.5 years and 70 years [2] and is more common in women [3]. ES commonly affects the parotid gland, followed by the submandibular gland [3]. In a case report, a patient was treated with total parotidectomy. This helped relieve the symptoms of swellings around the gland; however, the patient continued to have discharge from the Stenson’s duct, suggesting that this is likely to be a disease of the salivary ducts rather than the gland itself. There are studies that show the effectiveness of antihistamine medication in the management of ES due to its neutralizing effect on eosinophilic infiltration [4]. The most widely accepted pathophysiological hypothesis of the disease suggests that it is based on an allergic process involving the salivary glands [1,5]. This theory is supported by the fact that it is commonly seen in patients with atopy, presence of peripheral eosinophilia and some improvement on withdrawing the offending trigger [1,5]. Also, eosinophilic sialodochitis is noted to have similarities with asthma; both diseases affect large ducts while sparing the parenchyma. Additionally, both conditions produce eosinophil-rich mucus plugs and feature eosinophilic infiltration into the mucosa and submucosa [1]. Alpha-streptococcus has been identified in the mucus plugs, leading to the possibility of bacterial infection as an alternate mechanism of pathophysiology [6].

Symptoms of ES are attributed to the development of the mucus plug, which reduces the drainage of the saliva from the gland. The mucus plug formation is a response to the allergic trigger [4]. Kim et al. noted allergic symptoms in two-thirds of the patients in their study [4]. Carey et al., in their study, noted that the most reported symptom was swelling (97%), followed by itching of the overlying skin (92%), salivary gland discomfort (84%) and ‘string-like’ mucus discharge from salivary duct orifices (76%) [7]. The symptoms could be unilateral or bilateral, developing within a few hours to days [2].

Table 2 lists the two criteria for diagnosing ES. Prompt improvement when treated with anti-allergic treatment should alert the clinician to consider a diagnosis of ES.

**Table 2:** Criteria for diagnosing ES ES: eosinophilic sialodochitis, IgE: immunoglobulin E, IgG4: immunoglobulin G4 Source: Baer et al. [1] and Carey et al. [7]

Baer et al. [1]	Carey et al. [7]
Recurrent paroxysmal swelling of the major salivary glands	Presence of eosinophils in aspirated ductal contents obtained by the technique described
Salivary duct mucus plugs containing numerous eosinophils	Intermittent swelling of at least one major salivary gland
Peripheral blood eosinophilia and elevated IgE level	Presence of at least one of the following additional symptoms: itching of the skin overlying the affected gland, pain in the affected gland and expression of string-like mucus plugs
Associated atopic disease	Exclusion of other causes of salivary gland swelling with eosinophils
Ductal dilatation and occasional focal narrowing of the major salivary gland ducts	Diagnostic criteria: All the above criteria need to be met to confirm the diagnosis.
Periductal eosinophil- and lymphocyte-rich inﬂammation and ﬁbrosis with associated reactive ductal epithelial cells	
Failure to satisfy the diagnostic criteria of IgG4-related disease	
Diagnostic criteria: Criteria 1 and 2 need to be fulfilled, or criteria 1 along with 6 and 7.	

In our patient, she fits in with the criteria 1,3, 6 and 7 of Baer et al. As mentioned earlier, elevated serum eosinophilia and IgE levels are key findings in ES. Baer et al. noted that 71% and 72% of the patients in their study had elevated serum eosinophilia and peripheral IgE levels, respectively [1]. Another hallmark finding in ES is the duct dilation and enhancement along the duct, which can be seen on a contrast CT and magnetic resonance imaging (MRI) [4,8]. Other modalities, such as sialography and ultrasound scans, can also be used to demonstrate the duct dilation [4,8]. Terminal duct biopsy helps to confirm the diagnosis as well. Pathological features seen in the biopsy for ES are abundant eosinophils and lymphocyte infiltration around the duct, degranulation of eosinophils, extensive fibrosis and scattered mastocytes [9]. The study conducted by Zheng et al. showed that these were safe procedures and could be coupled with duct dilatation [9].

Differential diagnosis includes Sjögren’s disease, eosinophilic granulomatosis with polyangiitis, Kimura disease, chronic sialodochitis and IgG4-related disease. Antibodies such as anti-Ro and anti-la are positive in Sjögren’s disease, along with symptoms of dry mouth and dry eyes [6]. Unlike ES, IgG4-related sialadenitis is associated with elevated levels of IgG4 and often results in severe interstitial fibrosis and dense lymphoplasmacytic infiltrates [5]. Salivary glands are spared in eosinophilic granulomatosis with polyangiitis, and historically, extravascular granulomas and eosinophilic infiltrates are noted [9]. In Kimura disease, patients present with asymptomatic swelling, which is likely due to a lymph node or subcutaneous lesion, which may be asymptomatic [6]. Chronic obstructive sialadenitis (COS) is differentiated from ES based on histopathological findings; ES is characterised by milder atrophy and fibrosis and the presence of IgE, while in COS, eosinophil infiltration, hyperplasia of the lymphoid follicles around the duct and mucous metaplasia of the ductal epithelium were more severe [8].

Management can be divided into conservative, medical and surgical based on the severity. Conservative management includes compressive massaging over the affected area and adequate hydration [10]. The use of high-dose antihistamines and steroids forms part of the medical management [10]. The case report by González et al. documented the use of benralizumab in the management of eosinophilic sialodochitis [11]. Given the underlying pathophysiology of the condition, this treatment approach is likely to gain further traction in the future. Surgical procedures include duct cannulation and irrigation with saline, sialodochoplasty and glandular resection [1]. Glandular resection is the only treatment that has been shown to be beneficial [1]. Carey et al. reported that 35% of patients showed subjective improvement with sialendoscopy washouts of the affected salivary glands with concurrent intraductal corticosteroid deposition [7].

Table 3 summarises the differences between IgG4 disease, Sjögren’s disease and eosinophilic sialodochitis.

**Table 3:** Differences between IgG4 disease, Sjogren’s disease and eosinophilic sialodochitis IgG4: immunoglobulin G4, IgE: immunoglobulin E

Category	IgG4 disease	Sjogren’s disease	Eosinophilic sialodochitis
Definition	Systemic fibroinflammatory condition with IgG4+ plasma cell infiltration affecting multiple organs, including salivary glands [12]	Chronic autoimmune disorder of salivary and lacrimal glands causing lymphocytic infiltration [13]	Rare, inflammatory condition mainly affecting salivary gland ducts, causing eosinophilic infiltration [5]
Aetiology	Immune-mediated, the exact cause is not known, associated with IgG4+ plasma cells [12]	Mediated by B-lymphocytes [13]	Hypersensitivity reaction, eosinophilic inflammation [5]
Affected areas	Pancreas, salivary glands (especially submandibular), lacrimal glands, others (kidneys and lungs) [12]	Mainly salivary and lacrimal glands [13]	Salivary gland ducts and mainly the submandibular and parotid glands [5]
Clinical features	Gland swelling, tumefactive lesions, painless mass occasionally [12]	Xerostomia and keratoconjunctivitis sicca, extraglandular manifestations [13]	Recurrent painful swelling of salivary glands [10]
Histopathology	Dense lymphoplasmacytic infiltrate rich in IgG4+ plasma cells, storiform fibrosis and obliterative phlebitis [12]	Lymphocytic infiltration around ducts and destruction of acini [14]	Eosinophilic infiltration, large ducts with mucus plugs and periductal fibrosis [5]
Serology	Elevated serum IgG4 levels [12]	Positive anti-Ro/SSA and anti-La/SSB antibodies [13]	Peripheral eosinophilia in blood, raised serum IgE [1]
Imaging findings	Diffuse gland enlargement [12]	Parotid gland enlargement [13]	Dilated ducts with mucus plugs and irregular ducts [5]
Treatment	Corticosteroids, immunosuppressants [12]	Symptomatic (artificial tears, saliva substitutes), immunomodulators in severe cases [13]	Corticosteroids, anti-allergic therapy and sometimes surgical duct drainage [10]

## Conclusions

This case report aims to highlight and raise awareness of ES. Management includes conservative, medical and surgical strategies, as previously outlined. Additionally, emerging therapies, such as biologics, show promise and may play a significant role in future treatment; however, further comprehensive clinical trials are necessary to establish their efficacy.
